# Comparison of Predicted Exercise Capacity Equations and the Effect of Actual versus Ideal Body Weight among Subjects Undergoing Cardiopulmonary Exercise Testing

**DOI:** 10.1155/2013/940170

**Published:** 2013-04-03

**Authors:** H. Reza Ahmadian, Joseph J. Sclafani, Ethan E. Emmons, Michael J. Morris, Kenneth M. Leclerc, Ahmad M. Slim

**Affiliations:** ^1^Cardiology Service, Brooke Army Medical Center, 3551 Roger Brooke Drive, San Antonio, TX 78234-6200, USA; ^2^Pulmonary/Critical Care Service, Brooke Army Medical Center, 3551 Roger Brooke Drive, San Antonio, TX 78234-6200, USA

## Abstract

*Background*. Oxygen uptake at maximal exercise (VO_2_ max) is considered the best available index for assessment of exercise capacity. The purpose of this study is to determine if the use of actual versus ideal body weight in standard regression equations for predicted VO_2_ max results in differences in predicted VO_2_ max. *Methods*. This is a retrospective chart review of patients who were predominantly in active military duty with complaints of dyspnea or exercise tolerance and who underwent cardiopulmonary exercise testing (CPET) from 2007 to 2009. *Results*. A total of 230 subjects completed CPET on a bicycle ergometer with a male predominance (62%) and an average age of 37 ± 15 years. There was significant discordance between the measured VO_2_ max and predicted VO_2_ max when measured by the Hansen and Wasserman reference equations (*P* < 0.001). Specifically, there was less overestimation when predicted VO_2_ max was based on ideal body weight as opposed to actual body weight. *Conclusion*. Our retrospective analysis confirmed the wide variations in predicted versus measured VO_2_ max based on varying prediction equations and showed the potential advantage of using ideal body weight as opposed to actual body weight in order to further standardize reference norms.

## 1. Introduction

The determination of functional capacity to perform maximal exercise is one of the intended goals of any form of stress testing. Cardiopulmonary exercise testing (CPET) offers two specific advantages over conventional stress testing. During conventional testing, the degree of effort can be measured in several ways including subject report of volitional fatigue, ratings of perceived exertion, the percentage of predicted heart rate achieved, and the interpretation of the provider who is supervising the test. Another advantage of CPET is the direct measurement of maximal oxygen consumption as a measure of functional capacity, referred to as VO_2_ max.

The objective of this study was to compare published reference values for VO_2_ max based on ideal versus actual body weight to determine the effect on interpretation of maximal exercise during CPET.

## 2. Methods

### 2.1. Study Protocol and Oversight

The study is a retrospective review of a series of CPET data initially utilizing predicted VO_2_ max using the Jones 1983 reference equation: Male:  VO_2_ (L/min⁡) = 4.2 − (0.032∗age), and Female: VO_2_ (L/min⁡) = 2.6 − (0.014∗age).

Maximum VO_2_ was recalculated using different prediction equations and ideal versus actual body weight, respectively. Physicians trained in the interpretation of CPET determined if interpretation of maximal exercise differed using the various prediction equations (Jones et al., 1985; Hansen et al., 1984; Wasserman et al., 1999) as well as ideal versus actual body weight, respectively, as follows.

 Jones et al., 1985 [2]:
   VO_2_ (L/min⁡) = 0.046 (ht) − 0.21 (age) − 0.62 (sex) − 4.31,
 Hansen et al., 1984 [3]:
   Male: VO_2_ (L/min⁡) = wt  ∗(50.75 − (0.37∗age))/1000,
   Female: VO_2_  (L/min⁡) = (wt + 43)∗(22.78 − (0.17∗age))/1000,
 Wasserman et al., 1999 [4]:
   Male: VO_2_ (L/min⁡) = wt∗(50.72 − (0.372∗age))/1000,
   Female: VO_2_ (L/min⁡) = (wt + 42.8)  ∗(22.78 − (0.17∗age))/1000.

### 2.2. Data Collection

All CPET studies were performed in the Brooke Army Medical Center Pulmonary Function Laboratory beginning in January 2007 through December 2009. The study group primarily consisted of active duty military being evaluated for dyspnea or exercise intolerance. Studies were performed on a graded exercise test using an incremental protocol on a cycle ergometer, and patients performed a maximal exercise test until limited by fatigue or symptoms. Oxygen saturation was monitored with the LifeStat 1600 pulse oximeter (Physio-Control; Redmond, WA), and 12-lead electrocardiograph monitoring was accomplished via the Marquette 2000 during the test. Blood pressures were taken before the test and immediately upon completion of exercise. All participants were exercised using a standard protocol with increases in resistance of 25 watts every minute and were asked to continue exercising until exhaustion or limited by symptoms. During the entire warm-up, exercise, and recovery phases of the test, expired gas analysis was performed through the 2900 Series Metabolic Cart (Sensormedics; Yorba Linda, CA). Gas analysis measurements included oxygen consumption (VO_2_), carbon dioxide production (VCO_2_), tidal volume (TV), respiratory rate (RR), and minute ventilation (VE).

### 2.3. Statistical Analysis

Data are presented as mean ± SD. Demographic comparisons between genders were analyzed by a two-tail Student's *t* test. Actual measured VO_2_ max was compared to predicted VO_2_ max between all methods using a one-way ANOVA with Holm-Sidak post hoc test. Clinical agreement between algorithms for VO_2_ max using actual versus ideal body weights using limit of VO_2_ max ≤ 84% predicted maximums for nominal data was assessed using Cohen's kappa, and the McNemar test was employed to test discordance. *P*  values < 0.05 were considered significant. Regression analysis was employed to assess the strength of association between VO_2_ max predictors and actual VO_2_ max measurements, and a Bland-Altman test was employed to assess agreement throughout the range of predicted VO_2_ max for each algorithm.

## 3. Results

### 3.1. Baseline Patient Characteristics

**Figure 1:**
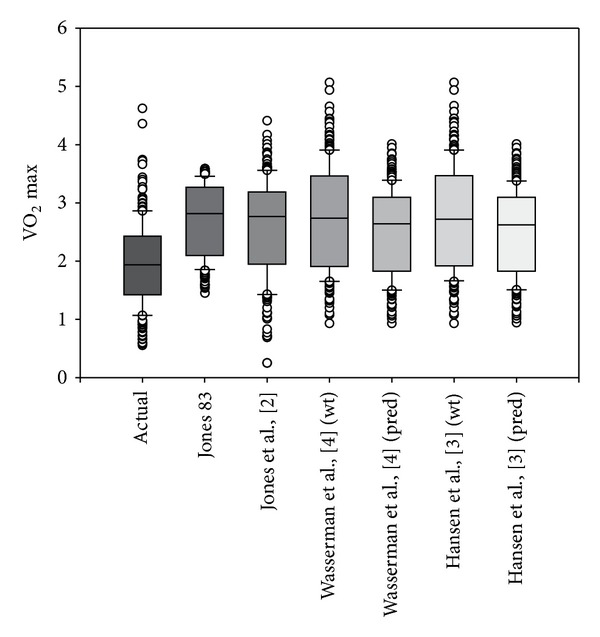
Figure indicates significant overestimation of predictors of VO_2_ max compared with actual measured VO_2_ max in this population. Significant differences among test (*P* ≤ 0.001).

**Figure 2:**
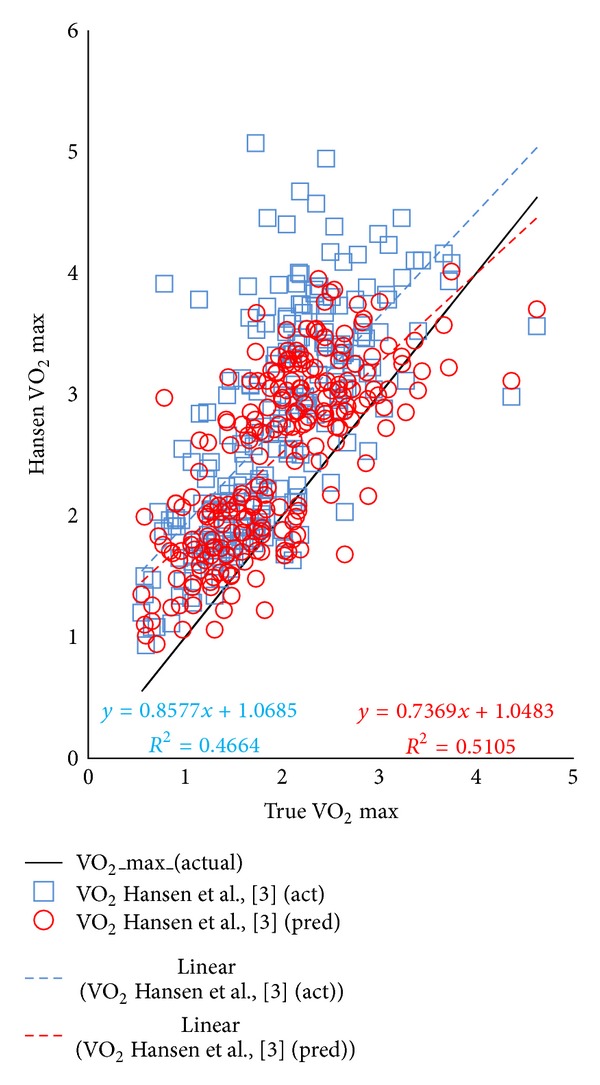
Comparison among regression lines for the Hansen algorithm using either actual or ideal body weights to predict VO_2_ max.

**Figure 3:**
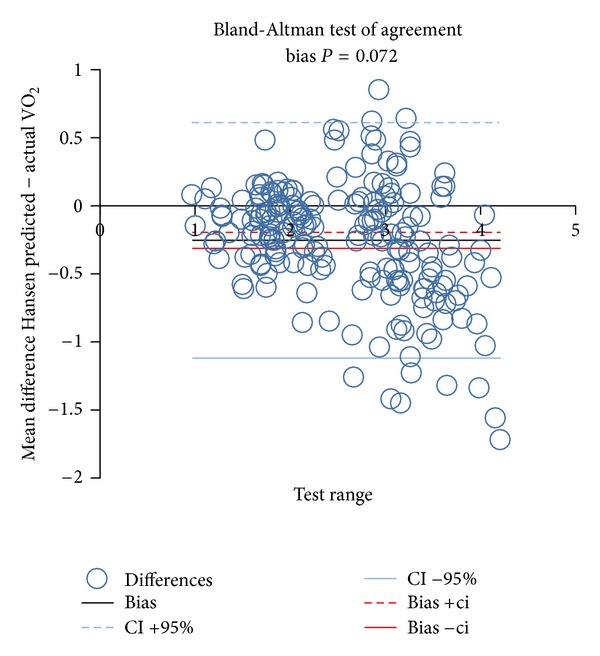
Illustration of Bland-Altman test of agreement between Hansen predicted VO_2_ max using ideal versus actual body weight in calculations.

**Figure 4:**
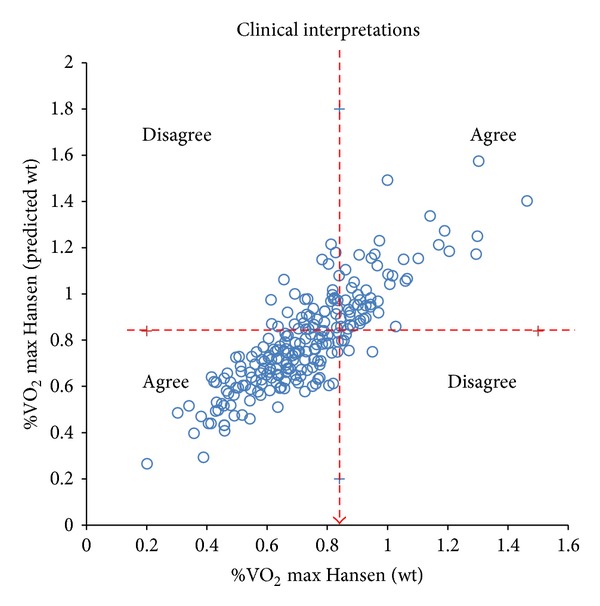
Presentation of the level of agreement between Hansen algorithm using actual versus ideal body weights to predict VO_2_ max ≤ 84%.

The study population consisted of 230 subjects with male predominance (62%) and a mean age of 37 ± 15 years.  Table 1 illustrates differences among genders with the population.  Figure 1 illustrates the marked variance among all VO_2_ predictive equations (*P* < 0.001), regardless of whether ideal body weight was used or not, as well as significant overestimation of predicted VO_2_ max compared with actual measured VO_2_ max in this population.  Figure 2 compares regression lines for the  Hansen algorithm using either actual or ideal body weights to predict VO_2_ max. Although *R* ^2^ was greater when using ideal body weight, the discordance of the estimates of true VO_2_ max, using ideal or actual body weights, was greater when VO_2_ max was low. Figures 3 and 4 indicate only a moderate agreement between Hansen algorithms using actual versus ideal body weights to predict VO_2_ max ≤ 84%. Although 80.8% of time results agreed (kappa = 0.566), there was significant discordance (19.2%,   *P* < 0.001) between tests.

## 4. Discussion

VO_2_ max reflects the product of cardiac output and the arteriovenous oxygen difference at peak exercise. Clinically, it is usually expressed as a percentage of predicted since it is believed to be more appropriate for intersubject comparisons as opposed to the standardization by body mass [1]. Because this is a weight-indexed value, differences in weight alone can impact the calculation irrespective of other objective factors. This is illustrated by the observation that obese patients have lower VO_2_ max results than those of normal weight due to the fact that adipose tissue is relatively metabolically inactive. The measurement of VO_2_ max is influenced by many factors to include age, sex, body size and composition, and level of aerobic training. Consequently, different prediction equations can yield different predicted VO_2_ values based on which variables are used in the calculations.

According to recommendations made by the American Thoracic Society/American College of Chest Physicians (ATS/ACCP) in a statement on CPET, the two most widely used sets of references values, Jones et al. [2] and Hansen et al. [3], should be used clinically. Wasserman et al. published as well a different set of reference values used for VO_2_ max in addition to the ATS/ACCP endorsed references mentioned earlier [4]. At least one study has demonstrated that different sets of maximal reference values can have significant impact on interpretation of CPET results [5].

The ATS/ACCP guidelines address the issue of peak VO_2_ prediction based on weight and acknowledge the absence of standardization regarding the best index of body size. They acknowledge the known miscalculations of VO_2_ max in obese patients. They allude to recommendations made by several experts about referencing VO_2_ to fat-free weight (FFW) and believe that this index has the added advantage of accounting for gender differences in VO_2_ max. However, the ATS/ACCP stopped short in making this recommendation since the routine measurement of FFW would be difficult to implement in most conventional exercise laboratories. The ATS/ACCP therefore recommends that VO_2_ max be expressed as an absolute value and as a percentage of the predicted value. Maximum VO_2_ should also be referenced to body weight (in kilograms) and/or height in the formatting of the report so that the impact of body size on exercise results is readily recognized [6].

Several experts have examined the applicability of body size and the interpretation of VO_2_ max. Buskirk and Taylor made the observation that VO_2_ max was more closely relevant to fat-free weight (FFW) than to total body weight. FFW may not be related to level of conditioning. They stressed the importance of calculating VO_2_ max in relation to lean body mass to avoid misclassification of obese patients [2]. Hansen and associates studied 77 ex-shipyard workers, one-third of whom were obese, defined as weight greater than 120% of expected for height. In this population, he proposed that height should be used with age and sex as predictors of VO_2_. His theory was tested using the formulas of Bruce and coworkers who first showed the relationship between height and weight in a sedentary middle-aged male population [3]. They used height to estimate normal weight and used the normalized weight in all those above this value. In only 2 of 77 subjects did the measured VO_2_ differ widely from the predicted VO_2_. Maximum VO_2_ was poorly predicted if actual weight was used in their obese population.

A recent study by Sill et al. examining CPET in a similar normal population of military personnel (mean age of 25.4 ± 4.3 years, body mass index of 24.4 ± 2.8, and percent body fat of 21.3 ± 6.1) found only a slight decrease in the predicted normal VO_2_ max to 82% predicted [7]. In a 1974 study in which 710 healthy, active duty Air Force personnel underwent maximal exercise testing, the authors published a regression equation used to predict VO_2_ max. However, the study population included only men, and the regression equation only factored in age, making no adjustments for height or weight. Another study evaluating exercise capacity in a military population included 1,514 male and 375 female active duty military personnel and reported VO_2_ max mean values of 51 and 37 mL/kg/weight/min for males and females, respectively [8].

In our study population, predicted VO_2_ max, when indexed to weight, was overestimated compared to measured VO_2_ max regardless of the predictive equation used. However, there was less overestimation when predicted VO_2_ max was based on ideal body weight (IBW) as opposed to actual body weight.

One of the limitations of this chart review is that approximately 70% of the study population failed to achieve 84% of predicted VO_2_ max. This could have been attributed to true pathology, decreased exercise capacity, obesity, or merely not being pushed to peak exertional capacity. The latter seems like the most plausible explanation for at least a portion of the subjects since evaluation of heart rates revealed that 31% of the study population also failed to achieve 84% of their target heart rate. Another important limitation of this study is that the study population included symptomatic subjects who were not an exclusively healthy group of young volunteers. This again highlights the need for a set of population-based norms for CPET evaluation and interpretation. Furthermore, physical fitness impacts the correlation among the reference equations, and the fact that only approximately one-third of men and women in our subgroup analysis met Air Force standards for fitness could be skewing our results.

Despite the previous limitations, this study is unique since comparing predicted to measured VO_2_ max using the variety of known prediction equations has never been done previously. Our retrospective analysis confirmed the wide variations in predicted versus measured VO_2_ based on varying prediction equations and shows the potential advantage of using ideal body weight as opposed to actual body weight in order to further standardize reference norms. It also illustrates the need for having population-specific reference norms for the most relevant and accurate interpretation of cardiopulmonary exercise testing.

## Figures and Tables

**Table 1:** Demographics gender variations.

Column 1	Male (*n* = 142)	Female (*n* = 88)	*P* value
Age (yrs)	36 ± 14	40 ± 16	0.049
Height (cm)	176.9 ± 8.2	163.9 ± 7.9	<0.001
Weight (Kg)	89.4 ± 18.3	72.3 ± 13.8	<0.001
Ideal weight (Kg)	79.0 ± 6.6	64.1 ± 5.3	<0.001
BMI (Kg/M^2^)	28.6 ± 5.6	27.1 ± 5.7	<0.001

## Abbreviations

CPET: Cardiopulmonary exercise testing
RER: Respiratory exchange ratio
VCO_2_: Carbon dioxide production
VO_2_: Oxygen consumption
VO_2_ max: Maximum oxygen consumption
ATS: American Thoracic Society
ACCP: American College of Chest Physicians
TV: Tidal volume
RR: Respiratory rate
VE: Minute ventilation
FFW: Fat-free weight.
